# Editorial: Adaptive optics for *in vivo* brain imaging

**DOI:** 10.3389/fnins.2023.1188614

**Published:** 2023-03-31

**Authors:** Cristina Rodríguez, Martin J. Booth, Raphaël Turcotte

**Affiliations:** ^1^Department of Biomedical Engineering, Yale University, New Haven, CT, United States; ^2^Department of Engineering Science, University of Oxford, Oxford, United Kingdom; ^3^Kingdom Supercultures, Brooklyn, NY, United States

**Keywords:** adaptive optics, brain, intravital imaging, optical aberrations, microscopy

Optical microscopy enables the non-invasive investigation of the dynamic structure and function of living organisms, providing the necessary spatial resolution to resolve cellular and subcellular components. In neuroscience, this unique ability has been translated into imaging the activity and connections of individual neurons and their processes in the living brain. The highly heterogeneous nature of biological tissues, however, causes refractive aberrations to the light used by optical microscopes, compromising the image brightness and resolution and limiting the imaging depth, ultimately holding back our understanding of many biological phenomena of interest. To address the challenge associated with imaging heterogeneous tissues, adaptive optics actively shapes the wavefront of light and compensates for the optical aberrations introduced by biological specimens, enabling subcellular resolution imaging at depth (Booth, 2014; Ji, 2017; Rodríguez and Ji, 2018). This Research Topic, focused on adaptive optics for intravital brain imaging, highlights recent advances in adaptive optics and its applications to different optical imaging modalities.

Super-resolution microcopy techniques are especially powerful tools as they enable the visualization of proteins and nucleic acids within intact cells while overcoming the fundamental resolution limits imposed by diffraction. Imaging whole cells or tissue specimens, however, remains a major challenge for super-resolution methods, where factors such as the background fluorescence and the aberrations induced by the sample affect the localization precision and largely limit the achievable resolution and penetration depth (Liu et al., 2020). In the work by Hung et al., the authors perform 3D single-molecule localization microscopy (SMLM) in tissues by combining adaptive optics (AO), *in situ* 3D point spread function (PSF) calibration, and 3D single objective lens inclined light sheet (SOLEIL) microscopy (Hung, 2022)—an approach based on an oblique light-sheet for achieving optimal optical sectioning and reducing the background fluorescence. To correct for the aberrations introduced by the optical system and the sample, the authors implement a model-based wavefront sensorless approach. The authors show the performance of AO-SOLEIL in different samples including fixed Drosophila brains, where aberration correction achieved a 2-fold improvement in the estimated axial localization precision when compared to using SOLEIL without AO or widefield microscopy with AO.

By using tightly-focused near-infrared laser pulses, two-photon (2P) fluorescence microscopy (2PFM) dramatically extends the imaging depth in tissues—when compared with one-photon-fluorescence-based techniques that use visible light for fluorescence excitation—and achieves optical sectioning due to the nonlinear excitation confinement (Denk et al., 1990). In the work by Matsumoto et al. the authors demonstrate an approach for high-resolution 2PFM at depth. To this end, the authors implement two resolution-enhancement methods that involved (1) using an azimuthally polarized vortex excitation beam and (2) modulating the amplitude distribution of the azimuthally polarized vortex beam using a multi-ring mask (Matsumoto et al., 2021). Assuming an average refractive index for the sample, the authors also calculate the expected spherical aberration caused by the refractive index mismatch between the immersion fluid and the sample, and correct for it using a spatial light modulator (SLM). The authors demonstrate the performance of this approach using different samples including a fixed mouse brain and find the lateral resolution to be approximately 20% higher than the diffraction limit obtained using a circularly polarized beam. The contribution from Cortes et al. showcases another application of AO in 2PFM, for investigating mitochondrial dynamics in the mouse cranial bone marrow and brain. Here, a sensor-based AO algorithm using a home-built Shack-Hartmann wavefront sensor was employed to correct for system aberrations, and a Zernike-mode-based sensorless AO algorithm approach was used for correcting low order tissue aberrations caused by the mouse cranial bone and brain tissue. An almost 2-fold increase in the fluorescence intensity and a reduction in the PSF width was found after aberration correction in the living mouse bone marrow, allowing the authors to better characterize mitochondrial health and the survival of functioning cells.

As the imaging depth is increased in tissues, the 2P excitation power needs to be increased exponentially to compensate for the scattered photons. As the power continues to be raised, the nonlinear excitation will no longer be confined to the focal region and the out-of-focus signal will approach that generated within the focal volume—at which point the maximum imaging depth for 2PFM is reached. Owing to the tighter signal confinement provided by the higher-order nonlinear excitation, and the reduced scattering experienced by longer excitation wavelengths, three-photon fluorescence microscopy (3PFM) extends the penetration depth in tissues beyond the limits of 2PFM (Wang and Xu, 2020). Since higher-order nonlinear processes are more strongly affected by optical aberrations (Sinefeld et al., 2015), implementing AO in such imaging modalities is especially important, as confirmed by recent work on adaptive optical 3PFM for *in vivo* brain imaging (Rodríguez, 2021; Streich, 2021). In the work by Sinefeld et al., the authors performed *in vivo* mouse brain imaging using 3PFM in combination with a model-based wavefront sensorless approach that used 55 orders of Zernike polynomials and a three-phase optimization algorithm. After aberration correction, the authors achieve close to diffraction-limited resolution in deep cortical layers and signal improvements of ≈4.5 ×. In the hippocampus, the authors found larger signal improvements (≈6.5 ×), as expected given the significant additional aberrations added by the overlaying layer of white matter.

To correct for wavefront distortions outside of the traditional regime dealing with lower-order wavefront aberrations, different methods can be applied. In the work by Sohmen et al., the authors discuss how turbidity can be quantified, and device a numerical model to emulate the effect of a 3D scattering medium of “tunable” transport mean free path on a light field by using a 2D phase mask displayed on an SLM. Using this model as well as experiments, the authors investigate the performance of a recently introduced approach called DASH (May, 2021)—Dynamic Adaptive Scattering compensation Holography—across different turbidity regimes and compare it to a modified version of the Continuous Sequential Algorithm (CSA) (Vellekoop and Mosk, 2008) which the authors call Amplified CSA (a-CSA). For low turbidity, DASH initially improves the 2P signal more slowly than a-CSA, while for higher turbidity the initial speed disadvantage of DASH disappears. From the second iteration onwards, DASH outperforms a-CSA regardless of the degree of turbidity. For both algorithms, the 2P signal enhancement grows for increasing degrees of turbidity. The authors showed a 5-fold improvement in the 2P signal from microglia (after 3 iterations of DASH and correcting for 256 modes), 200 μm deep inside fixed brain tissue.

The contributions in this special issue highlight the important role that adaptive optical methods continue to play in a range of microscopy modalities including super-resolution and multiphoton microscopy. By canceling out the aberrations introduced by inhomogeneous tissues, such as the brain, adaptive optical methods improve imaging performance and enable high resolution imaging of tissues at depth. We envision aberration correction modules will become an indispensable add-on component to optical microscopes in many laboratories, opening new avenues for biological exploration. To this end, continued efforts to make such methods readily available and easy to implement to non-experts will be essential.

## Author contributions

RT was the overall editor of the Research Topic. CR wrote the editorial. All authors checked and confirmed the final version of the editorial. All authors contributed to the article and approved the submitted version.
